# Annotation of *yellow* genes in *Diaphorina citri*, the vector for Huanglongbing disease

**DOI:** 10.46471/gigabyte.20

**Published:** 2021-05-24

**Authors:** Crissy Massimino, Chad Vosburg, Teresa Shippy, Prashant S. Hosmani, Mirella Flores-Gonzalez, Lukas A. Mueller, Wayne B. Hunter, Joshua B. Benoit, Susan J. Brown, Tom D’Elia, Surya Saha

**Affiliations:** ^1^ Indian River State College, Fort Pierce, FL 34981, USA; ^2^ Division of Biology, Kansas State University, Manhattan, KS 66506, USA; ^3^ Boyce Thompson Institute, Ithaca, NY 14853, USA; ^4^ USDA-ARS, US Horticultural Research Laboratory, Fort Pierce, FL 34945, USA; ^5^ Department of Biological Sciences, University of Cincinnati, Cincinnati, OH 45221, USA; ^6^ Animal and Comparative Biomedical Sciences, University of Arizona, Tucson, AZ 85721, USA

## Abstract

Huanglongbing (HLB), also known as citrus greening disease, is caused by the bacterium *Candidatus* Liberibacter asiaticus (*C*Las). It is a serious threat to global citrus production. This bacterium is transmitted by the Asian citrus psyllid, *Diaphorina citri* (Hemiptera). There are no effective *in planta* treatments for *C*Las. Therefore, one strategy is to manage the psyllid population. Manual annotation of the *D. citri* genome can identify and characterize gene families that could be novel targets for psyllid control. The *yellow* gene family is an excellent target because *yellow* genes, which have roles in melanization, are linked to development and immunity. Combined analysis of the genome with RNA-seq datasets, sequence homology, and phylogenetic trees were used to identify and annotate nine *yellow* genes in the *D. citri* genome. Manual curation of genes in *D. citri* provided in-depth analysis of the *yellow* family among hemipteran insects and provides new targets for molecular control of this psyllid pest. Manual annotation was done as part of a collaborative Citrus Greening community annotation project.

## Data description

### Introduction

Huanglongbing (HLB), also known as citrus greening disease, is the most serious threat to citrus sustainability [1–4]. HLB is caused by the bacterium *Candidatus* Liberibacter asiaticus (*C*Las) [5]. The Asian citrus psyllid, *Diaphorina citri* (NCBI:txid121845), is the vector for *C*Las (Hemiptera: Liviidae) [6]. There is no effective treatment for the pathogen, and pesticide application is the only mechanism to reduce psyllid populations [4]. However, current control strategies are not effective, so the development of novel mechanisms to reduce the psyllid vector population is necessary. Manual annotation of the *D. citri* genome to identify and characterize genes could identify targets for psyllid control treatments [7–9].

Here, we identified and characterized genes in the *yellow* gene family. The yellow proteins (dopachrome conversion enzymes, DCE) are involved in melanin biosynthesis [10]. Melanization is a critical function in insects [11]. Melanization can be triggered locally as an immune effector response, in which melanin is synthesized and cross-linked with other molecules in injured areas, resulting in the death of invading pathogens and hardening of the wound clot [12]. Melanization is also essential for cuticle sclerotization or tanning, which leads to hardening of the insect exoskeleton [13], and the prevention of moisture loss [10]. To develop gene-targeting or gene-suppressing treatments for yellow proteins, accurate gene sequences need to be established, annotated, and basic expressional details provided. Here we describe the *yellow* genes of *D. citri* using a combination of genome annotation and expressional differences [14] based on previously conducted RNA-seq studies.

### Context

**Figure 1.**
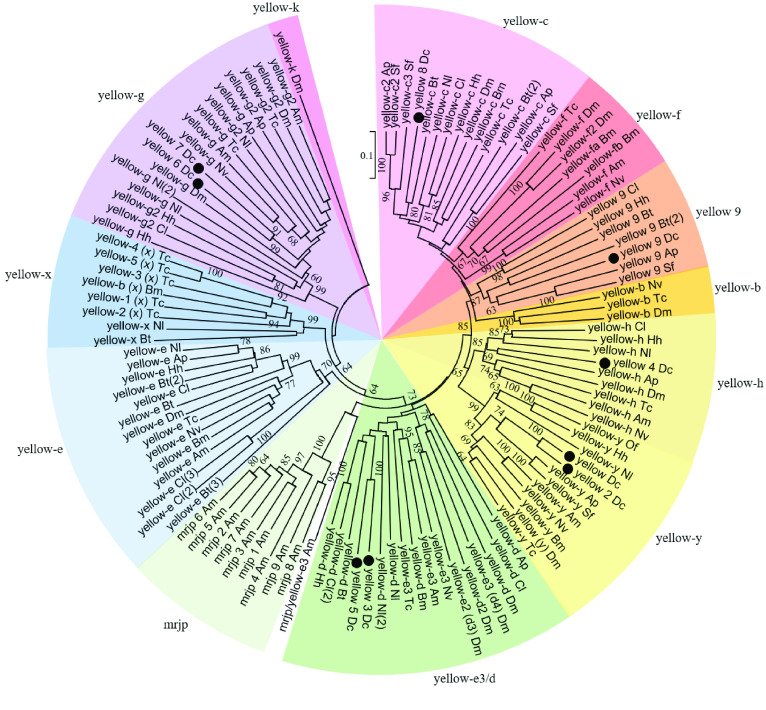
The *yellow* gene family. A neighbor-joining phylogenetic tree was generated from annotated *D. citri* (*Dc*) yellow protein sequences and the protein sequence of all predicted *yellow* genes from the insects *Acyrthosiphon pisum* (*Ap*), *Apis mellifera* (*Am*), *Tribolium castaneum* (*Tc*), *Drosophila melanogaster* (*Dm*), *Bombyx mori* (*Bm*), *Sipha flava* (*Sf*), *Bemisia tabaci* (*Bt*), *Nilaparvata lugens* (*Nl*), *Cimex lectularius* (*Cl*), *Halyomorpha halys* (*Hh*). Also included are sequences from *Nasonia vitripennis* (*Nv*) and *Oncopeltus fasciatus* (*Of*). Bootstrap analysis was performed with 1000 replicates. Values greater than 60 are shown at nodes. *D. citri* sequences are identified with a dot. Color coding indicates specific yellow clades. NCBI accession numbers are shown in Tables 2 and 3.

The *yellow* genes are of ancient lineage, as evidenced by the presence of *yellow*-like genes in several bacterial species. However, there is no evidence that these genes exist in the complete genome sequences of the worm *Caenorhabditis elegans* or the yeast *Saccharomyces cerevisiae*. This suggests that they may have been lost from many lineages and may now be largely restricted to arthropods [15]. While functional assignments have not yet been made for every member of this family, research suggests that a role in melanization may be conserved for several yellow family members [11]. Duplications, as well as losses, are apparent in the *yellow* gene family, and phylogenetic analysis shows that *yellow* family expansion is associated with insect diversification [11]. Previous studies have shown that the *yellow-y, -c, -d, -e, -f, -g,* and *-h* genes were present prior to divergence of the hemimetabolous and holometabolous insects; however, some of these ancestral *yellow* genes are lost in specific insect lineages [11]. The most notable case of *yellow* lineage duplication is the entire major royal jelly protein (mrjp) family, which forms a distinct cluster within the yellow family phylogeny and seems to be restricted to certain species of bees (Figure 1) [15]. Here we describe the *yellow* genes of the Asian citrus psyllid, *Diaphorina citri*. Because of the multiplicity of *yellow* genes discovered in *D. citri* and the inconsistency of ortholog names, phylogenetic analysis was performed to properly classify these genes. This was followed by examining expression differences based on previously available RNA-seq datasets. Based on these results, we discuss possible functions of the *yellow* genes identified in *D. citri*.

## Methods

**Figure 2.**
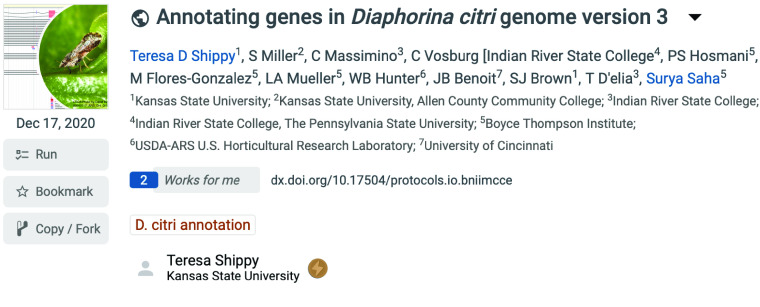
Protocols.io protocol for psyllid genome curation [20]. https://www.protocols.io/widgets/doi?uri=dx.doi.org/10.17504/protocols.io.bniimcce

**Table 1:** Evidence for gene annotations, including MCOT transcriptome sequences if applicable.

Gene	Identifier	MCOT	*de novo* transcriptome	Iso-seq	RNA-seq	Ortholog
*yellow* (*y*)	Dcitr06g10150.1.1	MCOT10626.0.MT	X	X	X	
*yellow 2* (*y2*)	Dcitr03g06860.1.1		X	X	X	
*yellow 3* (*d*)	Dcitr07g07190.1.1	MCOT21608.0.CT	X		X	
*yellow 4* (*h*)	Dcitr07g07210.1.1	MCOT05770.2.CC	X	X	X	X
*yellow 5* (*d*)	Dcitr11g08710.1.1	MCOT00471.2.CO	X		X	X
*yellow 6* (*g*)	Dcitr02g17890.1.1	MCOT15143.1.CC	X	X	X	X
*yellow 7* (*g*)	Dcitr11g06750.1.1	MCOT19435.0.CO	X	X	X	X
*yellow 8* (*c*)	Dcitr01g01210.1.1		X	X	X	X
*yellow 9*	Dcitr01g05760.1.1	MCOT08915.0.MM	X		X	

The *D. citri* genome was annotated as part of a community-driven manual curation project [9] with an undergraduate focus [16]. Protein sequences of the yellow family were collected from the National Center for Biotechnology Information (NCBI) protein database [17] and were used for a BLAST search of the *D. citri* MCOT (Maker (RRID:SCR_005309), Cufflinks (RRID:SCR_014597), Oases (RRID:SCR_011896), and Trinity (RRID:SCR_013048)) protein database [14]. MCOT protein sequences were used to search the *D. citri* genomes (version 2.0 and 3.0) [18]. Regions of high sequence identity were manually annotated in Apollo version 2.1.0 (RRID:SCR_001936), using *de novo* transcriptome and MCOT gene predictions, RNA-seq, Iso-seq, and ortholog data as evidence to determine and validate proper gene structure (Table 1). The gene models were compared with those from other hemipterans for accuracy and completeness. A neighbor-joining phylogenetic tree of *D. citri* yellow protein sequences, along with various related orthologs, was created in MEGA version 7 (RRID:SCR_000667) using the MUSCLE (RRID:SCR_011812) multiple sequence alignment with p-distance for determining branch length and 1000 bootstrap replicates [19]. A more detailed description of the annotation workflow is available (Figure 2) [20].

**Figure 3.**
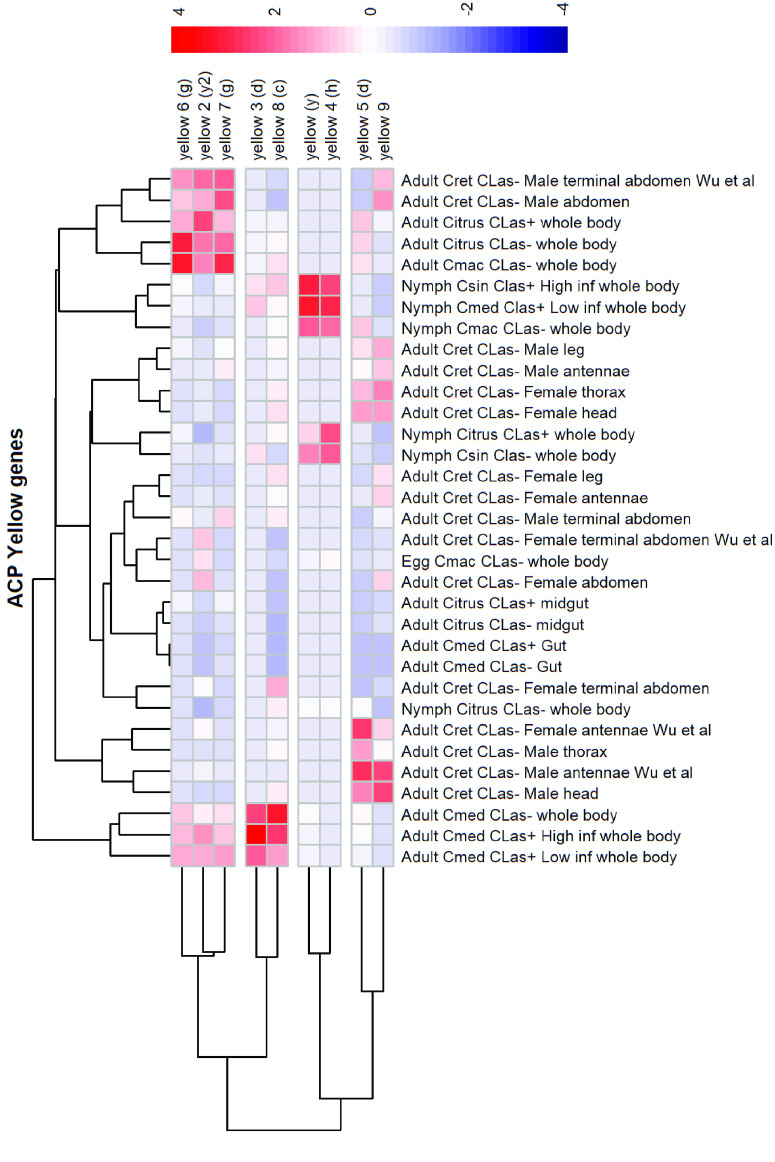
Comparative expression levels of the *D. citri* yellow-y proteins in infected vs. uninfected *D. citri* insects grown on various citrus varieties. Expression data were obtained using the Citrus Greening Expression Network [14]. RNA-seq data for psyllids were obtained from NCBI BioProject’s PRJNA609978 and PRJNA448935 in addition to published data sets [8, 23–26]. Citrus hosts are abbreviated as Csin (*C. sinensis*), Cmed (*C. medica*), Cret (*C. reticulata*) and Cmac (*C. macrophylla*).

**Figure 4.**
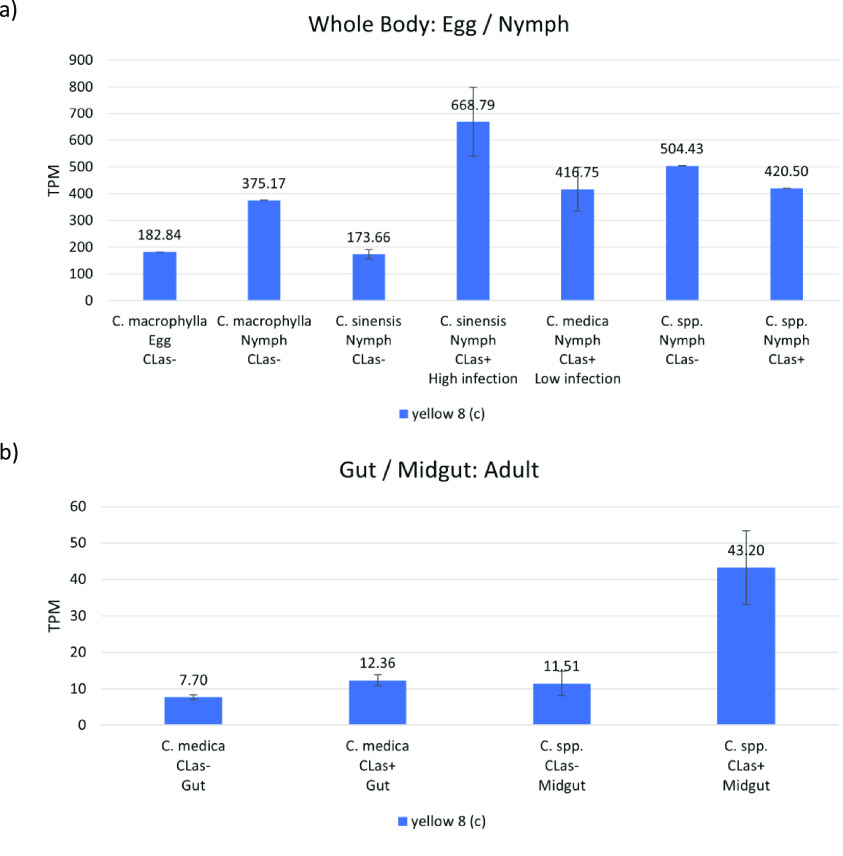
Comparative expression levels of the *D. citri* yellow 8 (*c*) proteins in uninfected *D. citri* insects grown on various citrus varieties. Values are represented in transcripts per million (TPM). Expression data obtained using the Citrus Greening Expression Network [14]. (a) Comparative expression levels in the whole body of *D. citri* egg and nymphs. Eggs and nymphs raised on *Citrus macrophylla* [8] and nymphs raised on *Citrus spp.* [25] are single replicate data. RNA-seq data were sourced from insects raised on *C. sinensis* and *C. medica* (NCBI BioProject PRJNA609978). (b) Comparative expression levels in the gut [24] and midgut [26] of *D. citri* adults.

**Figure 5.**
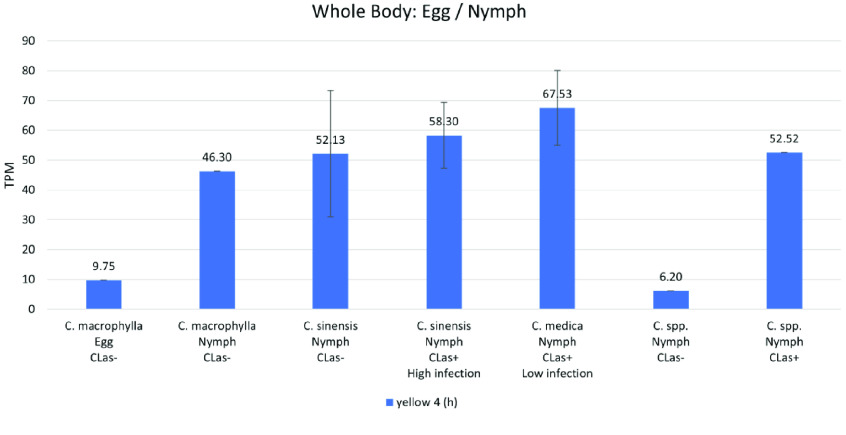
Comparative expression levels of the *D. citri* yellow 4 (h) proteins in eggs, nymphs and whole body infected and uninfected *D. citri* adults grown on various citrus varieties. Values are represented in transcripts per million (TPM). Eggs and nymphs raised on *Citrus macrophylla* [8] and nymphs raised on *Citrus spp.* [25] are single replicate data. RNA-seq data were sourced from insects raised on *C. sinensis* and *C. medica* (NCBI BioProject PRJNA609978). Expression analysis was performed using the Citrus Greening Expression Network [14].

**Figure 6.**
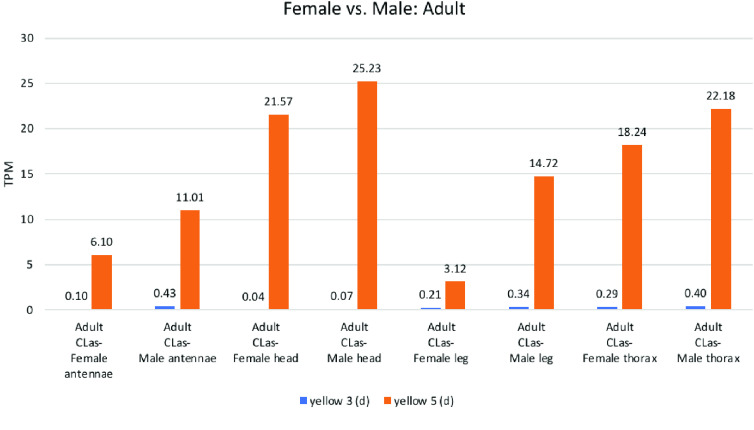
Comparative expression levels of the *D. citri* yellow-d proteins in female and male *D. citri* insects grown on *C. reticulata*. Expression data were obtained using the Citrus Greening Expression Network [14]. These samples from psyllid tissues have a single replicate and are from NCBI BioProject PRJNA448935.

**Figure 7.**
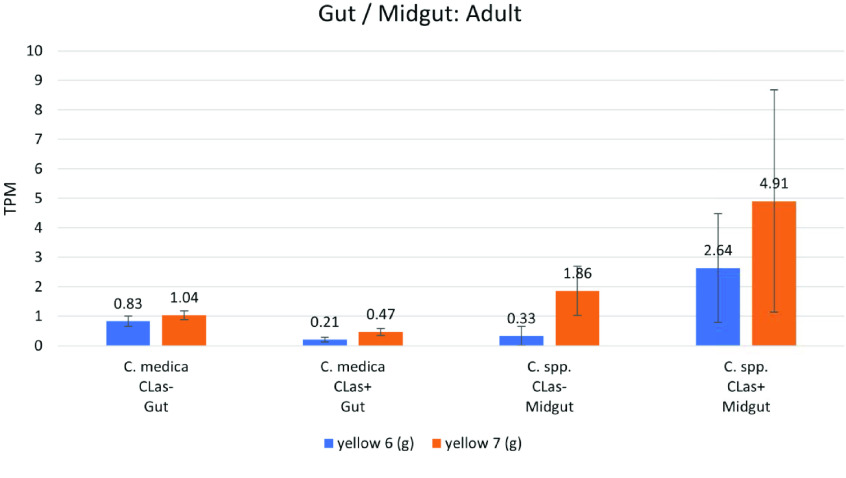
Comparative expression levels of the *D. citri* yellow 6 and yellow 7 (g) proteins in *D. citri* gut [24] and midgut [26] of infected and uninfected adult insects. Values are represented in transcripts per million (TPM). Expression data were obtained using the Citrus Greening Expression Network [14].

Accession numbers for the sequences used in this analysis can be found in Tables 1, 2, and 3. Comparative expression levels of yellow proteins throughout different life stages (egg, nymph, and adult) in *Candidatus* Liberibacter asiaticus (*C*las) exposed versus healthy *D. citri* insects was determined using RNA-seq data and the Citrus Greening Expression Network [14]. Gene expression levels were obtained from the Citrus Greening Expression Network [14] and visualized using Excel (RRID:SCR_016137) and the pheatmap package in R (RRID:SCR_016418) [21, 22]. Expression values for all samples discussed in this manuscript and visualized in Figures 3–7 are reported in Table 4.

**Table 2:** Non-hemipteran insect orthologs with accession numbers.

Gene	*Drosophila melanogaster*	*Tribolium castaneum*	*Bombyx mori*	*Apis mellifera*	*Nasonia vitripennis*
*yellow-y*	NP_476792.1	NP_001161919.1	NP_001037434.1	NP_001091693.1	NP_001154977.1
*yellow-b*	NP_523586.1	NP_001161777.1			NP_001154989.1
*yellow-c*	NP_523570.3	NP_001161778.1	NP_001037426.1		
*yellow-f* (*fa*)^†^	NP_524335.1	NP_001161780.1	NP_001037424.1	NP_001011635.1	NP_001154968.1
*yellow-f2* (*fb*)^†^	NP_650247.1		NP_001037428.1		
*yellow-h*	NP_651912.3	XP_008196499.1		NP_001091687.1	NP_001153394.1
*yellow-e*	NP_524344.1	NP_001161779.1	NP_001159615.1	XP_003249426.1	NP_001154985.1
*yellow-e2*	NP_650289.2				
*yellow-e3*	NP_650288.1	NP_001161913.1		NP_001091698.1	NP_001154982.1
*yellow-d*	NP_523820.2		NP_001037422.1		
*yellow-d2*	NP_611788.1				
*yellow-g*	NP_523888.1	ACY71060.1		XP_396824.1	XP_008210364.1
*yellow-g2*	NP_647710.1	NP_001161782.1		XP_006559006.1	
*yellow-k*	NP_648772.1				
*yellow-1* (*x*)^‡^		NP_001161783.1			
*yellow-2* (*x*)^‡^		NP_001161784.1			
*yellow-3* (*x*)^‡^		NP_001161785.1			
*yellow-4* (*x*)^‡^		NP_001161786.1			
*yellow-5* (*x*)^‡^		NP_001165862.1			
*yellow-b* (*x*)^‡^			NP_001037430.1		
*mrjp/yellow-e3*				XP_001122824.1	
*mrjp 1*				NP_001011579.1	
*mrjp 2*				NP_001011580.1	
*mrjp 3*				NP_001011601.1	
*mrjp 4*				NP_001011610.1	
*mrjp 5*				NP_001011599.1	
*mrjp 6*				NP_001011622.1	
*mrjp 7*				NP_001014429.1	
*mrjp 8*				NP_001011564.1	
*mrjp 9*				NP_001019868.1	

Accessions number for non-hemipteran insects used in phylogenetic analysis (Figure 1). XP identifiers are computer predicted models. ^†^ *Yellow-fa* and *-fb* are the names given specifically to *Bombyx mori*. ^‡^ *Yellow-x* is the name commonly used to refer to this group.

**Table 3:** Hemipteran orthologs with accession numbers.

Gene	*Acyrthosiphon pisum*	*Nilaparvata lugens*	*Sipha Flava*	*Bemisia tabaci*	*Halymorpha halys*	*Cimex lectularius*	*Oncopeltus fasciatus*
*yellow-y*	NP_001165848.1	XP_022184245.1	XP_025405013.1		XP_014294277.1		AMW91813.1
*yellow-c*	XP_008189794.1	XP_022204541.1	XP_025415761.1	XP_018916210.1	XP_014273608.1	XP_014260013.1	
				XP_018902711.1			
*yellow-c2*	XP_008189795		XP_025415762.1				
			XP_025421542.1				
*yellow-h*	XP_016656930.1	XP_022203506.1			XP_014273783.1	XP_014247137.1	
*yellow-e*	XP_001948479.1	XP_022204722.1		XP_018907407.1	XP_014292044.1	XP_024083966.1	
				XP_018906454.1		XP_014239663.1	
				XP_018907408.1		XP_014247778.1	
*yellow-d/e3*	XP_001942700.2	XP_022204983.1		XP_018916419.1	XP_014275903.1	XP_024083964.1	
		XP_022205442.1				XP_024083953.1	
*yellow-g*	XP_001944949.2	XP_022207733.1			XP_014292984.1		
		XP_022191720.1					
*yellow-g2*	XP_001945004.2	XP_022205647.1			XP_014292982.2	XP_014244848.1	
*yellow-x*		XP_022206946.1		XP_018912253.1			
*yellow 9*	XP_001946728.1		XP_025413197.1	XP_018905078.1	XP_014288983.1	XP_014255102.1	
				XP 018901286.1			

Accession numbers for hemipterans used in phylogenetic analysis (Figure 1). XP identifiers are computer-predicted models.

**Table 4:** Expression values (TPM) visualized in Figures 3–7.

Gene Name	*yellow* (*y*)	*yellow 2* (*y2*)	*yellow 3* (*d*)	*yellow 4* (*h*)	*yellow 5* (*d*)	*yellow 6* (*g*)	*yellow 7* (*g*)	*yellow 8* (*c*)	*yellow 9*
Gene ID	Dcitr06g10150.1.1	Dcitr03g06860.1.1	Dcitr07g07190.1.1	Dcitr07g07210.1.1	Dcitr11g08710.1.1	Dcitr02g17880.1.1	Dcitr11g06750.1.1	Dcitr01g01210.1.1	Dcitr01g05760.1.1
Egg C.mac *C*Las− Whole body	11.99	11.1	0.27	9.75	4.14	0	0	182.84	1.94
Nymph C.med *C*Las+ Low inf Whole body	277.12	5.89	23.24	67.53	5.72	2.98	3.72	416.75	0.49
Nymph C.sin *C*Las+ High inf Whole body	263.14	3.42	15.73	58.3	5.63	4.32	4.86	668.79	0.31
Nymph C.sin *C*Las− Whole body	153.79	4.14	18.8	52.13	4.97	2.63	2.95	173.66	0.53
Nymph C.mac *C*Las− Whole body	193.67	2.57	0.14	46.3	17.03	1.88	1.92	375.17	1.57
Nymph Citrus *C*Las− Whole body	32.81	0	0	6.2	9.78	0	0.29	504.43	0
Nymph Citrus *C*Las+ Whole body	77.7	0	0	52.52	6.57	4.01	0.67	420.5	0.24
Adult C.med *C*Las− Gut	0.06	0.76	0.15	0.88	0.21	0.83	1.04	7.7	0.08
Adult C.med *C*Las+ Gut	0.05	0.67	0.11	1.25	0.3	0.21	0.47	12.36	0
Adult C.med *C*Las+ High inf Whole body	10.89	17.9	96.66	0.73	8.8	13.47	17.05	1225.4	1.28
Adult C.med *C*Las+ Low inf Whole body	10.24	15.63	53.75	1.55	8.25	14.6	23.59	838.49	1.43
Adult C.med *C*Las− Whole body	26.67	10.11	59.97	1.44	10.19	11.3	13.59	1477.39	1.51
Adult C.mac *C*Las− Whole body	2.93	18.26	3.03	0.08	13.73	31.72	42.71	526.9	1.9
Adult Citrus *C*Las− Whole body	2.57	19.78	1.93	1.54	15.41	30.8	30.71	440.97	1.4
Adult Citrus *C*Las+ Whole body	2	24.29	2.71	0.45	16.75	14.24	19.45	313.19	2.24
Adult Citrus *C*Las− Midgut	0.12	2.32	0.08	0.28	1.84	0.33	1.86	11.51	1.34
Adult Citrus *C*Las+ Midgut	0.2	2.89	0.01	0.6	1.14	2.64	4.91	43.2	0.83
Adult C.ret *C*Las− Female abdomen	2.3	13.87	0.3	0.27	1.54	0	0.81	36.91	4.62
Adult C.ret *C*Las− Female antennae	3.43	5.73	0.1	0.59	6.1	0.07	1	394.88	4.45
Adult C.ret *C*Las− Female head	0.22	4.88	0.04	0.1	21.57	0	0	551.85	6.29
Adult C.ret *C*Las− Female leg	1.4	3.64	0.21	0.05	3.12	0	0.31	526.89	4.09
Adult C.ret *C*Las− Female terminal abdomen	6.01	7.65	0.22	0.11	0.2	0.44	0	736.67	0.9
Adult C.ret *C*Las− Female thorax	0.22	5.45	0.29	0.13	18.24	0	0	497.24	7.32
Adult C.ret *C*Las− Male abdomen	2.04	15.45	0.14	0.12	1.13	11.08	35.83	46.05	6.7
Adult C.ret *C*Las− Male antennae	5.47	5.72	0.43	0.31	11.01	2.12	12.49	301.88	5.14
Adult C.ret *C*Las− Male head	0.24	3.36	0.07	0.4	25.23	0	0.34	475.06	9.67
Adult C.ret *C*Las− Male leg	2.25	4.08	0.34	0.1	14.72	2.86	8.42	462.7	5.79
Adult C.ret *C*Las− Male terminal abdomen	2.74	5.64	0.24	0.22	1.33	6.55	15.52	512.54	2.41
Adult C.ret *C*Las− Male thorax	1.35	4.26	0.4	0.19	22.18	0.83	2.21	442.87	3.44
Adult C.ret *C*Las− Female antennae [23]	1.24	8.43	0.1	0.06	34.97	0	0.78	347.77	4.59
Adult C.ret *C*Las− Female terminal abdomen [23]	1.74	12.71	0.31	0.11	2.39	0	0.49	80.69	1.45
Adult C.ret *C*Las− Male antennae [23]	1.06	6.25	0.15	0.32	37.56	1.29	2.79	279.33	9.62
Adult C.ret *C*Las− Male terminal abdomen [23]	1.86	20.22	0.31	0.04	1.73	16.89	33.47	189.24	5.62

Comparative expression levels in transcripts per million (TPM) of the *D. citri* yellow-y proteins in infected versus uninfected *D. citri* insects grown on various citrus varieties. Expression data were obtained using the Citrus Greening Expression Network [14]. RNA-Seq data for psyllids were obtained from NCBI BioProject’s PRJNA609978 and PRJNA448935, in addition to published datasets [8, 23–26]. Citrus hosts are abbreviated as C.sin (*C. sinensis*), C.med (*C. medica*), C.ret (*C. reticulata*) and C.mac (*C. macrophylla*).

### Data validation and quality control

Manual gene annotation of the *D. citri* genome revealed the presence of nine *yellow* genes, each containing the major royal jelly protein (mrjp) domain conserved in this gene family. All nine of the genes were confirmed by at least four types of evidence, including RNA-seq and ortholog presence (Table 1). Phylogenetic analysis was conducted to determine the orthology of these *yellow* genes; the results coincide well with previous studies (Figure 1) [11, 27]. Based on this analysis, the *yellow* genes in *D. citri* comprise two *yellow-y* genes, two *yellow-d* genes, two *yellow-g* genes, one *yellow-h*, and one *yellow-c*, as well as one *yellow* gene (*yellow 9*) that seems to be a duplication unique to hemipterans, but is closely related to known *yellow-f* orthologs (Figure 1, Table 5).

Based on transcript and ortholog evidence, there are nine *yellow* genes in *D. citri* (Table 1). Each gene has been assigned an OGS3 identifier. Evidence from a *de novo* Oases or Trinity model from an independent transcriptome known as MCOT [9] was used to validate or improve our models. Illumina (RNA-Seq) reads were used to help create or validate our model. A *de novo* transcriptome was built from long RNA-Seq reads generated with Pacific Biosciences technology (Iso-Seq) and was used to help validate the exon structure of the models. These full-length transcripts allowed us to disambiguate noisy signals from short read RNA-seq data. Orthologous sequences from related insects and information about conserved motifs or domains were used to determine the final annotation. We used proteins from *Drosophila melanogaster* (*Dm*) [28], *Tribolium castaneum* (*Tc*) [29, 30], *Bombyx mori* (*Bm*) [31], *Apis mellifera* (*Am*) [32], *Nasonia vitripennis* (*Nv*) [33], *Acyrthosiphon pisum* (*Ap*) [34], *Nilaparvata lugens* (*Nl*) [35, 36], *Sipha flava* (*Sf*) [37], *Bemisia tabaci* (*Bt*) [38], *Halyomorpha halys* (*Hh*) [39], *Cimex lectularius* (*Cl*) [40], and *Oncopeltus fasciatus* (*Of*) [41].

Table 5 lists *yellow* gene family ortholog numbers in *A. pisum*, *A. mellifera*, *T. castaneum*, *D. melanogaster*, *B. mori*, *S. flava*, *B. tabaci*, *N. lugens*, *C. lectularius*, and *H. halys*. Genes manually annotated in the *D. citri* v3.0 genome are listed in the column labeled *Dc*. Phylogenetic analysis was performed using sequences in Tables 2 and 3 (Figure 1). *yellow-f* was not found in the genome or *de novo* transcriptome of *D. citri,* or in the hemipteran sequences used in this analysis. Instead, hemipterans formed a separate clade, which is labeled as *yellow 9* here (Table 5, Figure 1).

**Table 5:** Gene copy numbers in *D. citri* and related arthropods.

Gene	*Dc*	*Ap*	*Am*	*Tc*	*Dm*	*Bm*	*Sf*	*Bt*	*Nl*	*Cl*	*Hh*
*yellow-y*	2	1	1	1	1	1	1	0	1	0	1
*yellow-b*	0	0	0	1	1	0	0	0	0	0	0
*yellow-f*	0	0	1	1	2	2	0	0	0	0	0
*yellow 9*	1	1	0	0	0	0	1	2	0	1	1
*yellow-c*	1	2	0	1	1	1	3	2	1	1	1
*yellow-h*	1	1	1	1	1	0	0	0	1	1	1
*yellow-d/e3*	2	1	1	1	4	1	0	1	2	2	1
*yellow-e*	0	1	1	1	1	1	0	3	1	3	1
*yellow-g*	2	2	2	2	2	0	0	0	2	1	2
*yellow-x*	0	0	0	5	0	1	0	1	1	0	0
*yellow-k*	0	0	0	0	1	0	0	0	0	0	0
*mrjp* ^†^	0	0	10	0	0	0	0	0	0	0	0

The *yellow* gene family ortholog numbers based on results of the phylogenetic analysis (Figure 1). *Dc* numbers represent the number of manually annotated genes in the *D. citri* v3.0 genome. ^†^Copy number in *Am* includes a predicted sequence from NCBI that groups with *mrjp* in phylogenetic analysis.

####  yellow-y

The gene *yellow-y* (also simply referred to as *yellow*) was the first example of a single gene mutation affecting behavior [13]. However, *yellow*, was initially identified because of its role in pigmentation, and was named for the loss of black pigment that gave mutant flies a more yellow appearance [42]. Recent studies suggest that the *Drosophila melanogaster yellow-y* and *ebony* gene together determine the pattern and intensity of melanization [43], and that the *yellow-y* gene may regulate the expression of *yellow-f* or other enzymes involved in melanization [10]. Many studies have also noted a role for *yellow-y* in the behavior and mating ability of *Drosophila*, such as changes in the structures used during mating in *yellow* mutants [13]. The *yellow-y* gene is present in most insect species as a single copy; however, both *yellow* and *yellow 2* in *D. citri* form a clade exclusively with known *yellow-y* orthologs, indicating a duplication event that seems to be unique to *D. citri* (Figure 1). These two *yellow-y* genes show inverse expression patterns in *D. citri*; that is, *yellow* (*y*) shows highest expression in the nymph, while *yellow 2* (*y2*) shows highest expression in the adult (Figure 3). The high expression of *yellow (y)* in nymphs correlates with research that found *yellow-y* to be abundant in *Drosophila* pupae when melanin is deposited in the adult cuticle [44]. On the other hand, *yellow 2* (*y2*) may be important in adult *D. citri* and should be studied further.

#### *yellow-c*, *-f*, *-b*, and *9*

No function has been directly identified for the *yellow* genes *yellow-b* or *yellow-c*. However, phylogenetic analysis reveals a close relationship between *yellow-b*, *yellow-c*, *yellow-f*, and *yellow 9* [11, 27] (Figure 1). In *Drosophila*, *yellow-f* and *yellow-f2* function as dopachrome conversion enzymes (DCE), which are important in melanin biosynthesis [43]. Interestingly, while most hemipterans seem to have one or more *yellow-c* genes, none form clades exclusively with known *yellow-f* or *yellow-b* orthologs (Figure 1). Instead, hemipteran genes are grouped into their own separate clade. A close relationship was observed, however, between *yellow-f* and *yellow 9*, which is supported by the presence of an *Acyrthosiphon pisum* sequence in the *yellow 9* clade, previously reported as grouping with *yellow-f* (Figure 1) [11]. The addition of several other hemipteran sequences may have helped align *A. pisum* more closely to the *yellow 9* orthologs. This distinctness of the hemipteran group is common among the other *yellow* genes in the tree; however, the association to a known ortholog is typically much clearer than is seen with *yellow 9*. More studies should be conducted to conclusively determine the identity of this hemipteran outlier.

Of all *yellow* genes in *D. citri*, *yellow 8* (*c*) shows the greatest expression levels and is most highly expressed in the adult whole body of *D. citri* insects reared on *Citrus medica* (Figure 3). There was a significant increase in expression in nymphs reared on *C*Las-positive *Citrus sinensis*, with 3.85-fold upregulation in whole nymphs (Figure 4a). The gene was also upregulated by 3.75-fold in the midgut of infected adult psyllids reared on *Citrus spp.* (Figure 4b). These results suggest an interaction between *yellow 8* (*c*) and pathogen infection, therefore warranting further investigations.

####  yellow-h

Transcripts of *yellow-h* show color-related expression patterns in some species, but the function of the encoded protein is poorly understood [11]. Phylogenetic analysis reveals that *D. citri* contains one *yellow-h* gene, *yellow 4* (Table 5, Figure 1). Expression data from *D. citri* shows the highest expression of this gene in the egg and nymph (Figure 3). This is consistent with previous research showing that mutations of *yellow-h* in larval *Vanessa cardui* led to death in pupal stages of development, suggesting that *yellow-h* could be important during insect development [27]. Furthermore, expression data revealed an 8.47-fold increase in *yellow-h* expression in *D. citri* nymphs reared on *Citrus spp.* and infected with *C*Las (52.52 TPM) versus uninfected nymphs (6.2 TPM) (Figure 5). This differential expression of *yellow-h*, coupled with the impact of mutations in pupal mortality, indicates that *yellow-h* could be a potential RNA interference (RNAi) target and warrants additional study in *D. citri*.

####  yellow-e3/d

Previous research has revealed that *yellow-d* shows red-specific expression in the butterfly *V. cardui*, and that the loss of *yellow-d* function not only affects melanin patterns, but also presumptive ommochrome patterns [27]. Phylogenetic analysis of the *yellow* genes annotated in the *D. citri* genome shows *yellow 3* and *yellow 5* in a clade with known *yellow-e3/d* orthologs (Figure 1). Expression of *yellow 3* (*d*) was highest in the whole body of adult psyllids reared on *Citrus medica*, while those reared on *C. macrophylla* or *C. spp*. showed low expression. Similarly, expression was highest in nymphs raised on *C. sinensis* and *C. medica*, with almost no expression in psyllids reared on other citrus species (Figure 3). In insects reared on *C. reticulata*, expression of *yellow 3* (*d*) was consistently close to zero TPM, while *yellow 5* (*d*), showed relatively high expression in the adult antennae, head, and thorax (Figure 6).

####  yellow-g

The function of *yellow-g* is currently not well understood; however, it is often present in duplicate in most insects (Figure 1, Table 5). *D. citri* contains two *yellow-g* genes, *yellow 6* and *yellow 7*, both of which are expressed more in adult males versus females raised on *C. reticulata* (Figure 3). The expression of both genes is relatively similar throughout the stages and tissues that have been assayed. Neither gene is expressed in the egg, and both show low expression levels in the nymph, with higher expression in adults. There is a notable upregulation of *yellow 6* (*g*), from undetectable in uninfected nymphs raised on *C. spp.* to 4.01 TPM in infected nymphs. There is also an upregulation of *yellow 6* (*g*) by 8-fold and *yellow 7* (*g*) by 2.64-fold in the midgut of infected versus uninfected adult psyllids reared on *C. spp.* (Figure 7). This effect may indicate an immune response and should be studied further as a possible RNAi target.

### Conclusion

The *yellow* gene family is a continuously evolving set of genes, with duplications and losses among insects [11]. Many of these genes are crucial in melanization, which is essential for insect survival in relation to development and immunity [10, 12]. Though the function of some yellow proteins remain poorly understood, identification of these proteins in the hemipteran, *D. citri*, provides a novel insect lineage for studies of insect evolution and biology. *D. citri* harbors a unique duplication of *yellow-y*, a gene that may affect cuticular hardening. Therefore, it could be a potential target for a *D. citri*-specific molecular control mechanism [13]. Expression data shows an inverse relationship between the two *yellow-y* genes, suggesting independent roles for these proteins during juvenile and adult stages (Figure 3). The *yellow 9* gene appears to be unique to hemipterans (Figure 1), and is a potential alternative to *yellow-f* in holometabolous insects, which encodes a dopachrome conversion enzyme (DCE) in *Drosophila* [43].

## Reuse potential

The manually curated gene models based on the highly contiguous version 3 genome, compared with previous assemblies, provide a novel resource for understanding psyllid biology for the citrus greening community. To improve the accessibility and usability of this data, it will be included in the Psyllid Expression Network [14]. This visualization and analysis tool includes public transcriptomics data for *Diaphorina citri* from multiple tissues, life stages, infection states and citrus hosts in an expression cube for comparative analysis. Future directed studies are required to confirm the role of *yellow-9*. Continued examination of the *yellow* gene family across arthropods, and especially in insect vectors like *D. citri*, provide novel and species-specific gene targets, potentially through the use of RNAi, to control psyllid populations and reduce the effects of pathogens, such as *C*Las, causing citrus greening.

## Data Availability

The data sets supporting this article are available in the *GigaScience* GigaDB repository [45].

## Editor’s note

This article is one of a series of Data Releases crediting the outputs of a student-focused and community-driven manual annotation project, curating gene models and – if required – correcting assembly anomalies, for the *Diaphorina citri* genome project [18].

## List of abbreviations

*Am*: *Apis mellifera*; *Ap*: *Acyrthosiphon pisum*; BLASTp: protein BLAST; *Bm*: *Bombyx mori*; *Bt*: *Bemisia tabaci*; *Cl*: *Cimex lectularius*; *C*Las: *Candidatus* Liberibacter asiaticus; DCE: dopachrome conversion enzyme; *Dc*: *Diaphorina citri*; *Dm*: *Drosophila melanogaster*; *Hh*: *Halyomorpha halys*; Iso-seq: Isoform sequencing; MCOT: Maker, Cufflinks, Oasis, Trinity; mrjp: major royal jelly protein; NCBI: National Center for Biotechnology Information; *Nl*: *Nilaparvata lugens*; CGEN: Citrus Greening Expression Network; RNAi: RNA interference; RNA-seq: RNA sequencing; *Sf*: *Sipha flava*; *Tc*: *Tribolium castaneum*; TPM: transcripts per million.
